# Analog Convolutional Operator Circuit for Low-Power Mixed-Signal CNN Processing Chip

**DOI:** 10.3390/s23239612

**Published:** 2023-12-04

**Authors:** Malik Summair Asghar, Saad Arslan, HyungWon Kim

**Affiliations:** 1 Department of Electronics, College of Electrical and Computer Engineering, Chungbuk National University, Cheongju 28644, Republic of Korea; 2 Department of Electrical and Computer Engineering, COMSATS University Islamabad, Abbottabad Campus, University Road, Tobe Camp, Abbottabad 22044, Pakistan; 3 TSY Design (Pvt.) Ltd., Islamabad 44000, Pakistan

**Keywords:** mixed-signal convolutional operation, analog multiplier, neural network accelerator, convolutional neural network, artificial intelligence, neuromorphic engineering

## Abstract

In this paper, we propose a compact and low-power mixed-signal approach to implementing convolutional operators that are often responsible for most of the chip area and power consumption of Convolutional Neural Network (CNN) processing chips. The convolutional operators consist of several multiply-and-accumulate (MAC) units. MAC units are the primary components that process convolutional layers and fully connected layers of CNN models. Analog implementation of MAC units opens a new paradigm for realizing low-power CNN processing chips, benefiting from less power and area consumption. The proposed mixed-signal convolutional operator comprises low-power binary-weighted current steering digital-to-analog conversion (DAC) circuits and accumulation capacitors. Compared with a conventional binary-weighted DAC, the proposed circuit benefits from optimum accuracy, smaller area, and lower power consumption due to its symmetric design. The proposed convolutional operator takes as input a set of 9-bit digital input feature data and weight parameters of the convolutional filter. It then calculates the convolutional filter’s result and accumulates the resulting voltage on capacitors. In addition, the convolutional operator employs a novel charge-sharing technique to process negative MAC results. We propose an analog max-pooling circuit that instantly selects the maximum input voltage. To demonstrate the performance of the proposed mixed-signal convolutional operator, we implemented a CNN processing chip consisting of 3 analog convolutional operators, with each operator processing a 3 × 3 kernel. This chip contains 27 MAC circuits, an analog max-pooling, and an analog-to-digital conversion (ADC) circuit. The mixed-signal CNN processing chip is implemented using a CMOS 55 nm process, which occupies a silicon area of 0.0559 mm^2^ and consumes an average power of 540.6 μW. The proposed mixed-signal CNN processing chip offers an area reduction of 84.21% and an energy reduction of 91.85% compared with a conventional digital CNN processing chip. Moreover, another CNN processing chip is implemented with more analog convolutional operators to demonstrate the operation and structure of an example convolutional layer of a CNN model. Therefore, the proposed analog convolutional operator can be adapted in various CNN models as an alternative to digital counterparts.

## 1. Introduction

Convolutional Neural Networks (CNNs) have proven helpful in various applications ranging from character recognition to object detection. However, neural network resource usage is soaring with the continuous increase in feature complexity. This makes the deployments of CNNs on the existing CPU/GPU infeasible due to their size and power requirements. Furthermore, various Internet of Things (IoT) applications of CNNs have scarce energy sources and thus require solutions to lower power consumption in order to ensure the longevity of the devices [1]. As a result, there is a trend towards exploring high-performance neural processing units or accelerators with low power consumption.

Recently, neuromorphic architectures have been developed upon non-von Neuman architecture that can emulate the biological human brain network. Compared with traditional CPU/GPU designs established upon von Neuman architecture, neuromorphic architectures often provide superior power efficiency and parallelism [2]. Recent presentations include several neuromorphic and accelerated systems that make use of SNN [3,4,5,6] and CNN architectures [7,8,9].

Computing convolutional operations in the digital domain involves multipliers and adders, i.e., the Multiply-and-Accumulate (MAC) operation. For concurrent processing, the number of multipliers required equals the filter size, which can result in large area consumption. Moreover, summing the outputs of these multipliers involves multiple cascaded adders. Thus, digital MAC units occupy a massive area with higher power consumption. This area and power constraint caused the researcher’s interest to drift towards finding the new paradigm of analog kernels for CNN, which can not only perform convolution but can also occupy significantly less area and consume less power. Therefore, exploring unconventional architectures for the MAC unit is necessary.

A swarm of recent studies has focused on developing accelerators for CNNs [10,11,12,13,14,15], which attempt to improve the area, power consumption, and delay. In addition, some researchers are exploring mixed-signal approaches for CNNs [12,15], where some are integrating the analog compute units directly with the image sensor [13,15].

A 3 × 3 analog Convolutional Unit (CU) is implemented in [12], which requires differential analog input for weights and image values. Similarly, the analog CU of [10] is not a good choice for directly replacing the conventional digital CUs as it requires additional DACs to convert the filters and image values to analog. An analog light-weight CNN integrated with a CMOS image sensor is presented in [13], capable of performing face detection. In [13], only a 2 × 2 switched-capacitor CU is realized, which can be inadequate for even slightly complex feature extraction applications. A mixed-signal cellular neural network accelerator is presented in [14], targeting MNIST and CIFAR-10 datasets. Reference [14], however, does not natively support filter sizes larger than 3 × 3. Moreover, the cellular structure of [14] is not compatible with fully connected layers.

This paper presents and implements a compact mixed-signal CNN processing chip in a 55 nm CMOS process. The proposed analog convolutional operator (ACO) is implemented for CNNs, comprising low-power MAC units, which directly expect digital inputs for weights/filter values and image pixels. A compact and low-power multi-channel analog convolutional operator unit (ACU) is proposed and implemented, consisting of three convolutional operators, a max-pooling circuit, and an ADC circuit to replace conventional digital processing elements inside the CNN. The proposed ACO can also be adapted to fully connected layers. Furthermore, an example convolutional layer based on a 3 × 3 convolutional kernel of a CNN model has been constructed in a CNN processing chip to illustrate the structure and functionality of the proposed ACU. This paper elucidates the architecture and design methodology for mixed-signal CNN processing chip implementation, elemental circuit designs, and simulation results. Firstly, in Section 2, the complete architecture of the mixed-signal CNN processing chip implementation is illustrated. Subsequently, in Section 3, the design and implementation of the underlying CMOS circuits for the proposed analog convolutional operator are presented and validated by simulations. Section 4 describes the complete implementation of the proposed mixed-signal CNN processing chip and simulation results. Finally, in Section 5, the overall performance is discussed and compared with other digital and analog CNN accelerators before a conclusion is drawn in Section 6.

## 2. Mixed-Signal Processing of Convolutional Neural Network

### 2.1. Architecture of Analog Convolutional Operator

**Figure 1:**
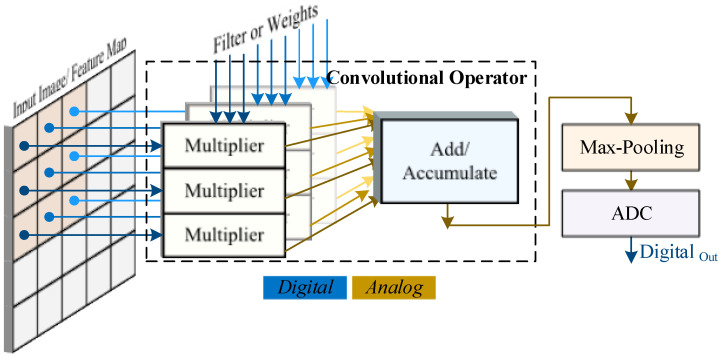
Mixed-signal block diagram of the architecture of the analog convolutional operator.

Figure 1 shows the overall structure and use of the mixed-signal CNN containing an analog convolutional operator (ACO), which can replace a conventional digital convolutional operator. The digital values of input image pixels and filter weights are directly applied to the ACO through a digital parallel interface. The ACO can perform MAC operations by containing *n* multiplier circuits for a convolutional layer with a filter size of $n=H_{filter}\times W_{filter}$. An accumulator circuit sums together the output of the *n* multiplier circuits. In addition, the 2 × 2 max-pooling layer is realized by a max-pooling circuit, which takes four convolutional result values as inputs and selects the maximum value as output. Finally, the result of the max-pooling is converted back to the digital domain using an ADC for further output feature map processing.

### 2.2. Multi-Channel Analog Convolutional Operator Unit

**Figure 2:**
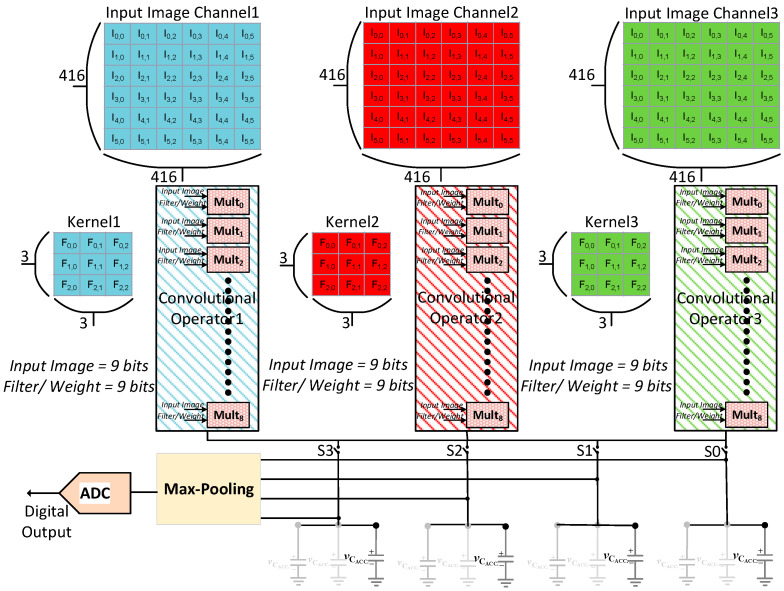
Multi-channel analog convolutional operator unit comprising three input channels.

Figure 2 illustrates an example architecture of a three-channel analog convolutional operator unit (ACU). It comprises three analog convolutional operators that replace a conventional digital processing element (PE) array used in conventional digital CNN chips. In Figure 2, the digital input data of three channels and corresponding filter weight parameters are fed independently to each convolutional operator. In this work, we use an implementation of each convolutional operator consisting of nine multipliers for realizing a 3 × 3 convolutional computation since it is most common in various CNN models. However, the proposed architecture is not limited only to a 3 × 3 structure. The outputs of the three convolutional operators are summed together and accumulated in an analog memory consisting of capacitors. The capacitors in the analog memory maintain the accumulated values in the form of voltage levels. In this work, we use an analog memory consisting of four capacitor pairs to store four convolutional computations for four neighboring 3 × 3 kernels on the input data.

The convolution results are immediately used to compute the analog max-pooling operations, which are often employed by convolutional layers in many common CNN models. The four output voltages of the capacitors are fed directly to the max-pooling circuit to compute a max-pooling operation of a 2 × 2 kernel. This work shows an example implementation of a 2 × 2 max-pooling circuit. The proposed max-pooling circuit can be easily extended to process various kernel sizes.

Finally, the output of the max-pooling operation is converted to digital value using a lower-power Successive Approximation Register (SAR) ADC. In the proposed analog convolutional operator unit, moving the max-pooling layer before the ADC and right after the four convolutional operations exploits the benefits of analog MACs while offering at least four times the reduction in analog-to-digital conversions. Moreover, this will inherently curtail the precision requirements of the system by discarding the small computational values. The proposed analog convolutional operator unit integrates the operations of a MAC, a max-pooling, and an ADC to perform one-shot calculations for the convolutional and max-pooling layers. Therefore, the proposed analog convolutional operator unit finds the advantages of area, power, energy, and speed.

## 3. Circuit Design of Analog Convolutional Operator

### 3.1. Analog Multiply-and-Accumulate Circuit

**Figure 3:**
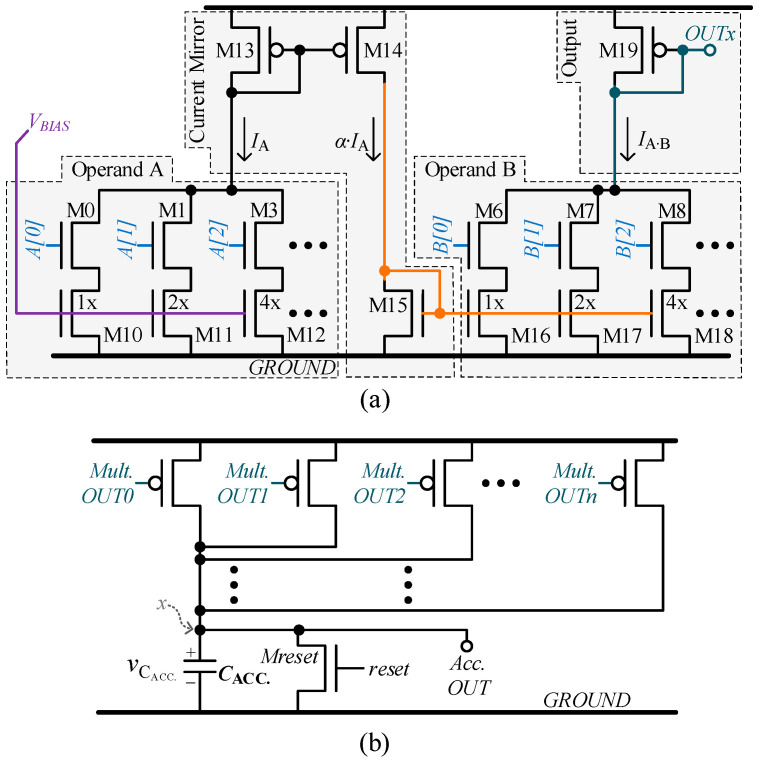
Analog MAC unit: (**a**) current steering DAC-based multiplier; (**b**) accumulator summing and integrating all the multipliers output currents.

The proposed analog convolutional operator aims to replace a digital convolutional operator while maintaining the input and output data in a digital domain. The digital inputs are converted to analog, and the analog outputs of the multiply-and-accumulate (MAC) circuit are converted back to digital. We propose a compact and low-power analog convolutional operator circuit consisting of several multipliers and an accumulator circuit. The multiplier circuit, designed for 4-bit input [16] and optimized and expanded to 8-bit input here, uses a pair of binary-weighted current steering digital-to-analog converters (DACs), which take the digital voltage values as inputs and produce current values as the output. Figure 3a illustrates the structure of the proposed multiplier. The multiplier tightly integrates two current steering DACs, each of which takes operand A and B, respectively. The first (left) DAC is supplied with a fixed bias voltage and generates an output current *I_A_*, which is proportional to the digital code provided by operand A. Next, a current mirror circuit is used to generate a bias voltage for the second (right) DAC based on the output of the first DAC. As a result, the current *I_A.B_* produced by the second (right) DAC is proportional to the product of operands A and B. Finally, the output current of the second DAC is converted to voltage using M19.

The accumulator circuit that follows the multipliers is shown in Figure 3b. The accumulator circuit converts output voltages from *n* multiplier circuits into corresponding currents. The outputs *OUTx* of *n* multipliers are connected to the nodes *OUT*0, *OUT*1, …, *OUTn* of the accumulator. These currents are summed together at node ‘x’, which is used to charge the accumulation capacitor *C_ACC_.* Before the start of computation, the accumulation capacitor is discharged through NMOS *M_reset_* by applying the *reset* signal. After accumulation, the expression for the final capacitor voltage can be derived from Equation (1).

(1) $$V_{C_{ACC.}}=\frac{Q}{C_{ACC.}}$$

Here, Q represents the charge stored in the capacitor while the inputs are applied and can be expressed as Equation (2).

(2) $$Q=\int_{0}^{T}I_{x}\cdot dt=I_{x}\times T$$

In Equation (2), $I_{x}$ represents the summed current, and T is the duration for which this current is applied (multiplier has valid inputs). Therefore, the final voltage on the accumulation capacitor can be expressed by Equation (3).

(3) $$V_{C_{ACC.}}=\frac{I_{x}\times T}{C_{ACC.}}$$

To avoid saturation or under-utilization of capacitor voltage, choosing a proper combination of $I_{x}$, T, and $C_{ACC.}$ is important. The current $I_{x}$ can be indirectly controlled using the bias voltage, while the duration of accumulation T can be determined by the digital controller described later. To ensure low power consumption, all the MOSFETs are kept at sub- or near-threshold gate voltages. This puts a constraint on the exploitable range of the bias voltage. The proposed MAC unit employs a configurable array of accumulation capacitors to achieve the adjustment of capacitance.

#### 3.1.1. Multiplier Circuit Design for Compactness

**Figure 4:**
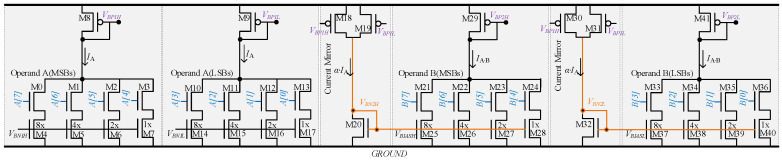
The 8-bit multiplier design constituted from two 4-bit current steering DAC circuits.

The area of the conventional binary-weighted DAC circuits increases exponentially as the resolution of inputs increases. In contrast, in the proposed multiplier circuit, to increase the input resolution to 8-bit for operands without increasing the area, a 4-bit current steering DAC is used as a fundamental building block. The proposed 8-bit multiplier circuit is shown in Figure 4. This design is derived from the two DACs of Figure 3a, where each DAC is further constituted from two 4-bit DACs. The first (left) DAC in Figure 4 is connected to the 4 MSBs and 4 LSBs of operand A, respectively. Similarly, the MSBs and LSBs of operand B are tied to the two 4-bit DAC units of the second (right) DAC.

**Figure 5:**
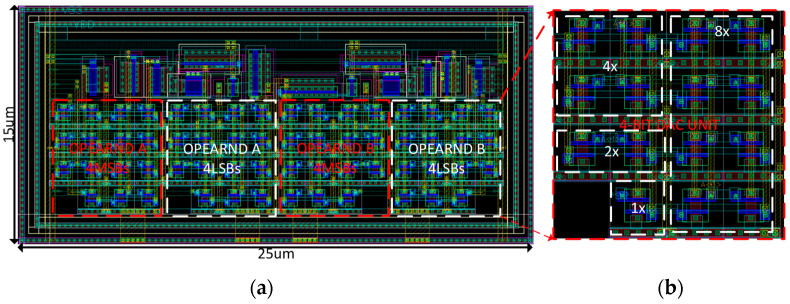
(**a**) The 8-bit multiplier layout design consisting of four 4-bit current steering DAC circuits; and (**b**) the layout design of a 4-bit DAC circuit.

The layout design of the 8-bit multiplier constituted from the four 4-bit DAC units is illustrated in Figure 5a. Each 4-bit DAC in the multiplier is constructed by placing 15 unit cells in a matrix structure to ensure better matching compared with a conventional binary-weighted DAC, which is depicted in Figure 5b. As a result, the proposed multiplier circuit provides a symmetric and compact design that occupies a small area of 375 μm^2^. Moreover, the increase in resolution linearly increases the area, which is in contrast to the exponential increase in area in a conventional binary-weighted DAC circuit.

#### 3.1.2. Simulation Results for the Multiplier

**Figure 6:**
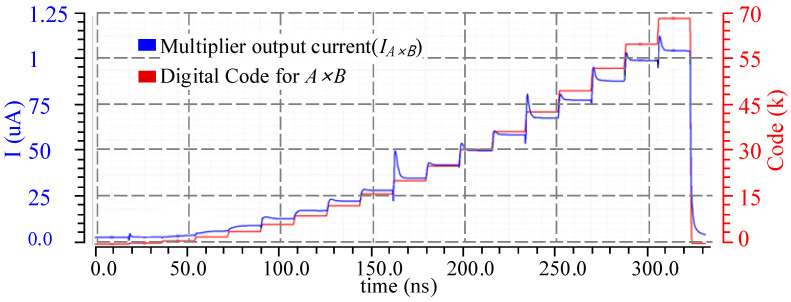
Simulation results for current steering DAC-based multiplier’s output current against the digital code.

The proposed 8-bit binary-weighted current steering multiplier was simulated to verify the operation, and the results are illustrated in Figure 6. Here, the incrementing digital input data, with a step size of 15, are applied, shared by both multiplier operands. The simulation shows the output current $I_{A\times B}$ of the multiplier (blue waveform) and the digital product results of operands A and B (red waveform). It can be observed that upon applying the digital input data, the output current rapidly transitions to a value that closely matches the product of operands A and B. In addition, to have low power consumption, all the MOSFETs used in the MAC unit have high threshold voltage, which offers low leakage and static current. Since the circuit deals with nano-amperes of current, any leakage would significantly impact the accuracy of the result. The proposed 8-bit multiplier consumes 1.44 μW of power to multiply two operands with a maximum product value of 255 × 255.

#### 3.1.3. Accumulator Circuit Design

The accumulator circuit in the proposed convolutional operator is designed using MOSFETs to steer the output current of the multiplier to be accumulated upon capacitors, as shown in Figure 3b. In the example circuit presented in this work, a four-capacitor array *C_ACC_* is implemented to store the accumulation value of four different input image pixel values convolved with filter values with stride movement value 1. The four capacitors in array *C_ACC_* accumulate, respectively, the current amount representing the four neighboring convolution results, which are passed over to the max-pooling circuit to select the maximum of the four convolution results. Each convolutional operation is conducted over a set of data in a 3 × 3 matrix of *C* channels out of the total input data channels, where *C* is set to three in our implementation in this work. For the first layer of the CNN, the input data matrix is the input image, while for other layers of CNN, the input data are the feature data produced by the previous layer. Firstly, the first convolution result is obtained as follows: The three ACOs of the proposed analog convolutional operator unit simultaneously convolve the three 3 × 3 filter kernels over the top-left 3 × 3 matrix selected from the input data with three input channels. The result of the first convolution is stored in the first capacitor in array *C_ACC_* in the form of voltage *V_ACC_*. For the second convolution, the 3 × 3 filter kernel shifts in the right direction over the input data by a stride value *S* and stores this convolutional result in the second capacitor in *C_ACC_*. In this work, we use a stride value *S* of 1. In a similar fashion, the 3 × 3 filter kernel moves down for the third convolution, and then it moves left for the fourth convolution. These results are stored in the third and fourth capacitors in *C_ACC_*, respectively. In the implementation of *C_ACC_*, the metal–oxide–metal (MOM) capacitors are employed to benefit from their higher capacitance density and linear current–voltage (CV) curve [17]. Each capacitor is implemented using configurable parallel capacitors, so each can be configured to have a size from 300 fF up to 700 fF in steps of 10 fF. Before accumulation, each capacitor’s voltage is reset to a reference voltage *V_ref_* of value 400 mV by a *reset* signal provided by a digital controller. Afterward, the digital controller generates a *start accumulation* signal that enables the current from the ACOs to accumulate in the respective capacitors of *C_ACC_*.

#### 3.1.4. Accumulator Circuit for Negative Values

In general, MAC operations of a convolutional layer of CNN models must handle the accumulation of both positive and negative MAC values. Conventional analog convolutional operators, however, do not provide efficient ways to compute the negative MAC values and accumulate the negative and positive MAC values into convolution results. In contrast, our proposed analog convolutional operator can handle both negative and positive MAC values as follows.

**Figure 7:**
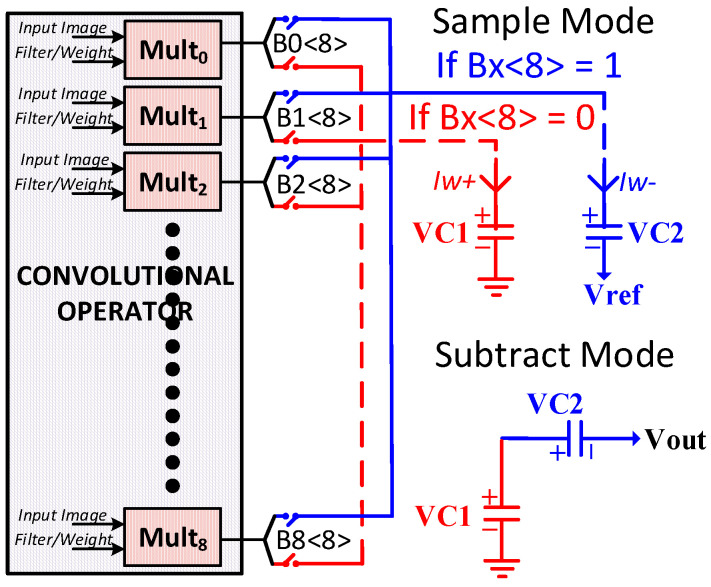
Subtraction of negative multiplications result from positive multiplications result.

The proposed MAC unit implements a charge-sharing technique to achieve the multiplication of negative values, as illustrated in Figure 7. Each of the four capacitors in array *C_ACC_* is split into a pair of two capacitors, each having a capacitance of 700 fF in the current implementation. The first capacitor (C1) of the pair accumulates the current *I_W+_* from the positive multiplication values. The second capacitor (C2) of the pair accumulates the current *I_W−_* from the negative multiplication values. For this purpose, a sign bit is added to the 8-bit value of operands to present the operands in a 9-bit signed magnitude format. The lower than 8-bit values are directly applied to the multiplier, while the 9-bit value (MSB), as a sign bit, is utilized for steering the multiplication currents either onto the positive capacitor (C1) or negative capacitor (C2). The operation comprises the sampling mode and subtracting mode. During the sampling mode, the equivalent currents *I_W+_* and *I_W−_* are accumulated as voltages *VC*1 and *VC*2, respectively, in capacitors C1 and C2 connected in parallel formation. During the subtracting mode, on the other hand, the connection of capacitors C1 and C2 is changed to series formation, and the charge sharing generates the subtraction result *V_out_* at the output,

*V_out_* = *VC*1 − *VC*2.

**Figure 8:**
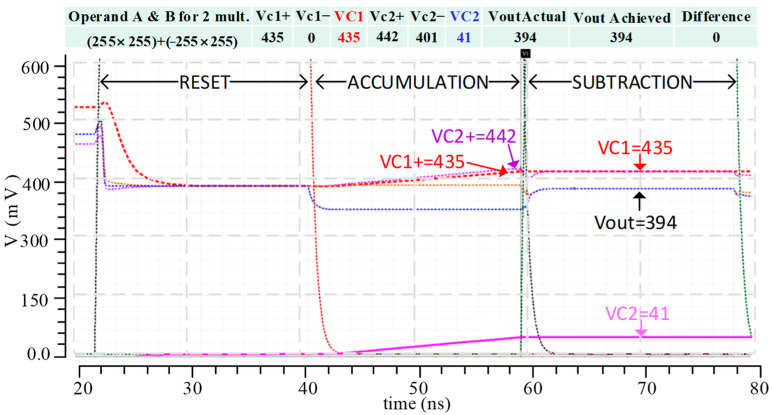
Simulation results illustrating the subtraction via charge sharing.

To explain the operation of the subtraction, Figure 8 illustrates the simulation result of subtracting *VC*1 and *VC*2, which are the voltage results from two multipliers. In this example, the first multiplier takes a maximum positive operand A and B value (255 × 255) to produce the highest output value. In contrast, the second multiplier takes a maximum negative and positive value (−255 × 255) to produce the lowest negative value. The subtraction example operates as follows. Firstly, the digital controller gives a *reset* signal, which discharges all the accumulation capacitors to the *V_ref_* value (400 mV). Secondly, the digital controller gives a *Start Accumulation* signal, which triggers the accumulation of currents in the capacitor pairs. As a result, the current *I_W_****_+_*** charges the capacitor voltage *VC*1 up to the equivalent *VC*1+, while *I_W_****_−_*** charges *VC*2 up to *VC*2+. Finally, the digital controller gives a *subtract* signal, which subtracts the two voltages between *VC*1 and *VC*2, and then generates an equivalent *V_out_*. The proposed analog MAC unit utilizes the existing accumulation capacitors to implement the charge-sharing technique for negative multiplication. Therefore, it does not require extra circuitry to implement the multiplication of the negative values, unlike the conventional methods of using complementary circuits.

### 3.2. Analog Max-Pooling Circuit

**Figure 9:**
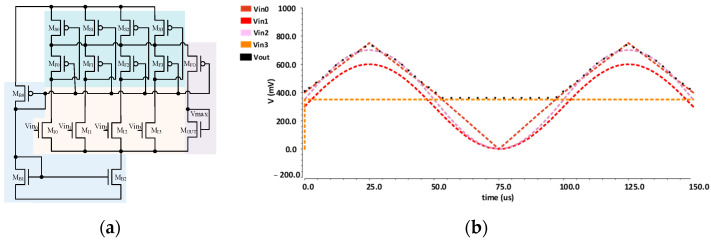
Analog max-pooling unit: (**a**) implemented voltage-mode max-voltage selection circuit; (**b**) simulation results for max-pooling circuit showing *V_out_* tracking maximum *V_in_*.

The proposed ACU employs a 12-bit SAR ADC to convert the convolution results to digital values. The ADC takes 14 clock cycles to complete an analog-to-digital conversion, which can limit the overall throughput of the ACU. To improve the throughput, we propose an analog max-pooling circuit that can be performed in the analog domain before conducting ADC. For example, in a max-pooling of size 2 × 2 with stride 1, an analog max-pooling can reduce the required conversion operations of the ADC by four times, reducing the energy consumption of the ADC by four times. The proposed max-pooling circuit is designed based on a voltage-mode max-voltage selection circuit, as shown in Figure 9a [18,19]. In this work, we implement the max-pooling circuit for a 2 × 2 pooling size since many CNN models employ a 2 × 2 max-pooling. It accepts four input voltages and provides an output voltage corresponding to the input voltage’s maximum voltage value. The four accumulation voltages of the four accumulation capacitors are applied to the inputs of the max-pooling circuit to perform one-shot computations. The simulation results are illustrated in Figure 9b, where the maximum voltage *V_out_* is indicated by a black curve.

### 3.3. Analog-to-Digital Converter

The proposed ACU intends to replace the digital convolutional operators while keeping the input and output memories unchanged; therefore, the output of the ACU must be converted back to digital for storing the outputs in memory and further processing them for the proceeding layers of CNN. For this purpose, a 12-bit Successive Approximation Register (SAR) ADC, introduced in [17], is employed by the ACU implementation presented in this work. To ensure minimal size and power consumption, the SAR ADC is implemented by further employing a split capacitor array DAC structure while eliminating the need for the sample and hold circuit by utilizing the accumulation capacitors to hold the input voltage. As a result, our implementation of the ADC achieves higher performance, reduces kick-back noise, provides rail-to-rail dynamic range, and significantly reduces area and power consumption. Furthermore, the complete implementation of the ACU also realizes auxiliary circuits, including an on-chip oscillator capable of generating clock signals up to 300 MHz and output buffers to drive observing outputs.

## 4. Implementation of Analog CNN Processing Chip

In this work, to demonstrate the performance and advantage of the proposed architecture, we implement a set of analog convolutional operators in a mixed-signal CNN processing chip. In this section, we first describe an implementation of a small ACU chip comprising three ACO circuits, and then present an implementation of a mixed-signal CNN processing chip consisting of eight ACU blocks.

### 4.1. Operation of On-Chip Digital Controller

**Figure 10:**
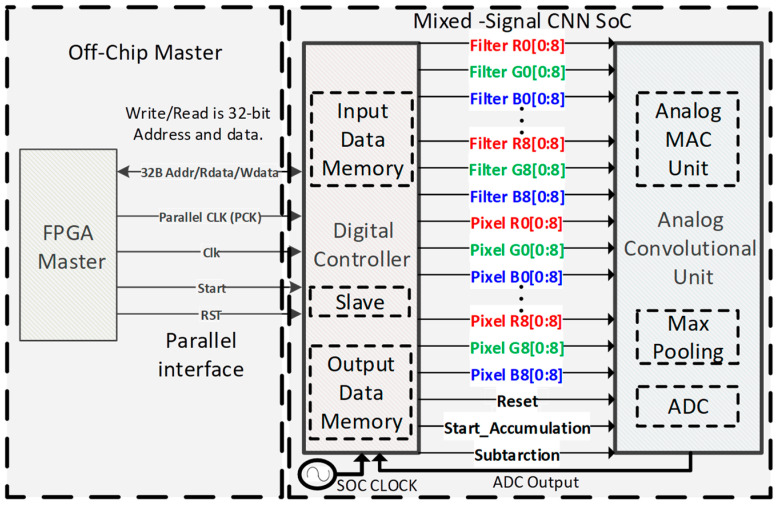
The block diagram of the 32-bit digital parallel interface for the mixed-signal CNN processing chip.

To prove the feasibility of the proposed ACU, a mixed-signal CNN processing chip is implemented. The proposed ACU is controlled by an on-chip digital controller, which triggers each step of the analog convolutional operations and the max-pooling operations, as illustrated in Figure 10. It also communicates with an external master processor through a 32-bit parallel interface to receive and transmit data. For each input of data, the digital controller takes a 3 × 3 image and filter weight data from the parallel interface and stores them in the input data memory, which are then sent to the ACU for convolutional computations. Upon each completion of convolution, the digital controller stores the ADC output in the output data memory. Once all convolutions are completed, it reads the convolution result data and transmits them to the external processor.

### 4.2. Fabrication of ACU Chip

**Figure 11:**
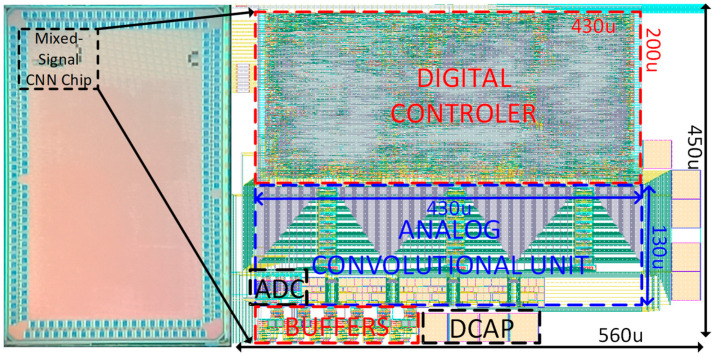
Micrograph and the demarcated layout of the ACU chip for mixed-signal CNN processing chip implementation.

The ACU chip consisting of the proposed analog convolutional operator unit and the digital controller is realized in a 55 nm CMOS process. Figure 11 illustrates the micrograph of the fabricated chip and demarcates the layout design of the ACU chip’s active area, which occupies a chip core area of 0.252 mm^2^. The analog convolutional operator unit comprises three channels of analog convolutional operators, a max-pooling unit, and a SAR ADC occupying the 0.0559 mm^2^ active analog core area of the chip. In addition, the ACU chip incorporates an oscillator and output buffers. The average power consumption measured from the ACU chip is 540 μW.

### 4.3. Simulation Results of ACU Chip

**Figure 12:**
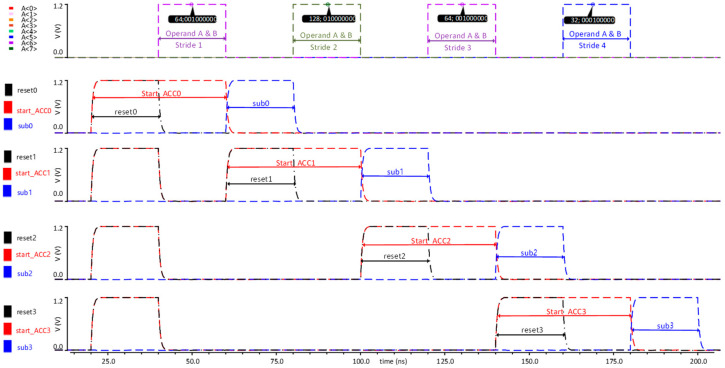
Timing diagram showing the control signals and the applied input signals for the two operands.

To elaborate the operations of the analog convolutional operator unit and digital controller, this section shows the simulation results using four continuous sets of convolutional, max-pooling, and ADC operations. Figure 12 illustrates the timing diagram for the input data and control signals. The analog convolutional operator unit operates on the digital input data and filter weights, which are the operands A [0:8] and B [0:8] for the MAC units. Three digital control signals for each accumulation capacitor pair in the capacitor array are applied to trigger the reset, start accumulation, and subtraction operations. Firstly, a *reset* signal discharges all four accumulation capacitors to the *V_ref_* value, which is configured to 400 mV in our experiment. Afterward, a *start accumulation* signal triggers the accumulation of the currents generated by the multiplier in the accumulation capacitors. During this period, the input data of both operands A and B for all 27 multipliers of three convolutional operators are kept valid. Finally, a *subtract* signal triggers the subtraction of the positive and negative accumulation voltages, as explained earlier in Section 3.1.4. During this period, the first accumulation capacitor provides the output voltage corresponding to the first convolutional operation. In the same fashion, the sequence of three control signals is repeated for all four accumulation capacitors one after another to perform four convolutional operations.

**Figure 13:**
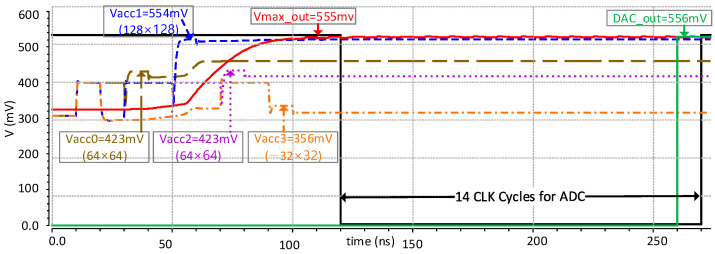
Simulation results verifying the operation of analog convolutional operator unit.

Figure 13 shows the simulation results of the analog max-pooling operations followed by analog convolutional operations. After the convolutional operation of 3 × 3, it performs a 2 × 2 max-pooling operation followed by an ADC operation. The example of Figure 13 applies the identical operands of A [8:0] and B [8:0] to all nine multipliers in each analog convolutional operator for the sake of simplicity. Each of the four convolutional operations takes as input 64 × 64, 128 × 128, 64 × 64, and −32 × 32, respectively. Upon receiving the first *start accumulation* signal, the multiplier produces a current value equivalent to the product of 64 × 64 and charges the respective accumulation capacitor *C_ACC_*, providing an equivalent voltage *V_ACC0_*. Afterward, upon receiving the *subtract* signal, the output voltage of 423 mV is obtained, which represents the first convolutional operation as indicated by the brown waveform. Similarly, the second convolutional operation for the input operands of A [8:0] = 128 and B [8:0] = 128 produces a resulting voltage *V_ACC_*_1_ of 554 mV as indicated by the blue waveform, which is equivalent to 128 × 128. For the third convolutional operation, the resulting voltage *V_ACC2_* of 423 mV is obtained (purple waveform), which is equivalent to 64 × 64. Lastly, for the fourth convolutional operation, the resulting voltage *V_ACC3_* of 356 mV is obtained (orange waveform), which is equivalent to −32 × 32. The four accumulation voltages corresponding to four convolutional operations are directly applied to the max-pooling circuit, which selects the maximum voltage and produces a *V_max_out_* of 555 mV (red waveform). Afterward, upon receiving a *start_ADC* signal (black signal), the ADC converts the max-pooling output into a 12-bit digital value. To verify the accuracy of the ADC, an ideal DAC is added to the simulation, which converts the digital value back to the analog value, resulting in 556 mV (green waveform). The error between the digital and analog values is as small as 1 mV. The SNDR and ENOB of the ADC are measured as 68.45 dB and an 11.07-bit value, respectively [17].

### 4.4. Architecture of Mixed-Signal CNN Processing Chip

**Figure 14:**
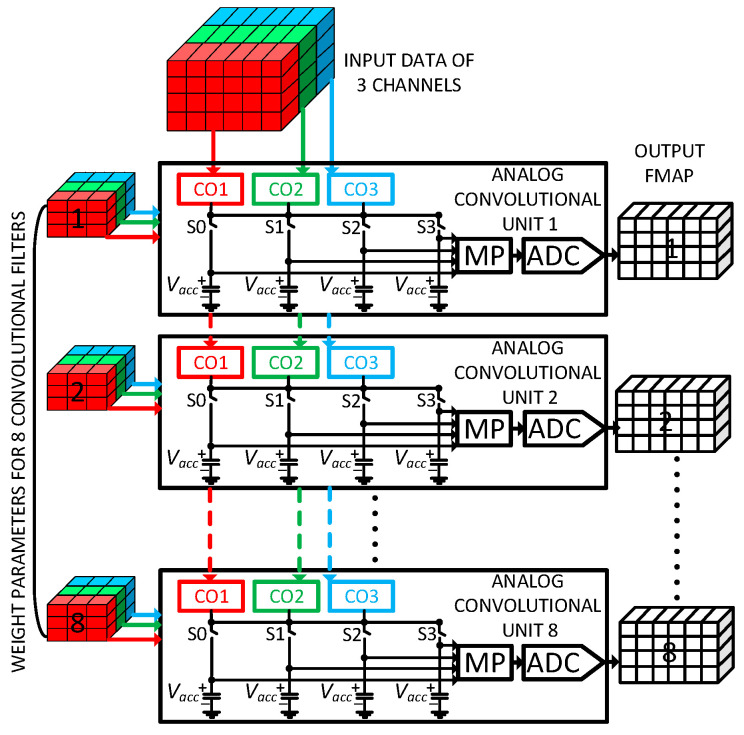
Overall architecture of an example mixed-signal CNN chip consisting of eight analog convolutional operator units with 3 input channels each.

In this work, to demonstrate the scalability of the proposed analog convolutional operator unit (ACU), we implemented a mixed-signal CNN processing chip consisting of eight ACU blocks. Figure 14 shows the overall architecture of the mixed-signal CNN processing chip that comprises eight ACUs, a common digital controller, a clock oscillator, and a high-speed interface for the host CPU. Each ACU contains three channels of convolutional operators, which can simultaneously perform a convolutional operation upon three channels of the input data and weight parameters and produce feature data for the corresponding output channel. Similarly, the eight ACUs can perform simultaneous convolutional operations for eight output channels upon receiving the following data for three input channels. The eight output channels, called convolutional filters, take the same three input channels using eight different weight parameters. The common digital controller shared by the eight ACUs provides input image and weight data to all the ACUs.

After convolutional computation, each of the eight ACUs calculates eight parallel max-pooling results and converts them to digital values using eight ADCs. Therefore, the proposed implementation can produce eight channels of concurrent output feature maps. The kernel size of the proposed ACU is not limited to 3 × 3, but it can be easily increased by increasing its number of MAC units. Therefore, the proposed analog convolutional operator unit is suitable for realizing various CNN models on hardware.

### 4.5. Example Implementation of Mixed-Signal CNN Processing Chip

**Figure 15:**
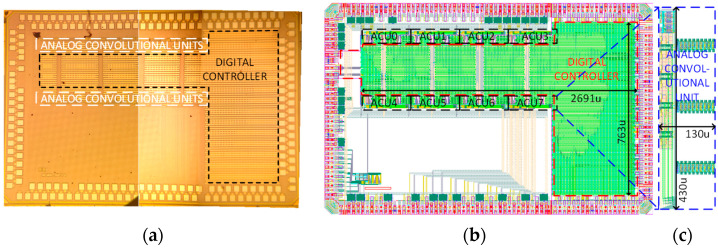
(**a**) The micrograph of the mixed-signal CNN processing chip, (**b**) the demarcated full chip layout, and (**c**) the zoomed-in structure of the one ACU.

The mixed-signal CNN processing chip is fabricated in a 55 nm CMOS process. Figure 15 lays out the micrograph of the fabricated chip and demarcates the layout of the active core area of the chip, which occupies a chip core area of 2.75 mm^2^. The compact design of the eight ACUs, which are tiled together with minimal routing, occupies only a very small area of 0.4784 mm^2^. The average power consumption of the mixed-signal CNN processing chip is as low as 1.4424 mW. The compact area and low power consumption of the CNN processing chip demonstrate the advantage that the proposed architecture can be easily extended to accommodate large-scale CNN models with a large number of convolutional filters and layers.

## 5. Performance Analysis

To demonstrate the performance and cost of the ACU chip in comparison with conventional architectures, we compare the implementation result of the ACU chip with an implementation of a digital neural network processing unit (NPU) [20] in Table 1. The NPU is implemented using the same 55 nm process technology as the proposed mixed-signal CNN. The NPU comprises 288 processing elements (PEs), each of which is composed of an 8-bit MAC operator. While the proposed ACU chip can be extended to cover the whole CNN model, we restrict our experiment only to the first layer of the CNN model for proof of concept. We consider an example CNN model called YOLOv2-tiny which consists of nine convolutional layers. For simplicity, we analyze the computation of only the first layer of the YOLOv2-tiny model. Since the proposed ACU chip comprises 27 MAC units with a 9-bit configuration for a three-channel 3 × 3 convolutional filter, we have downscaled the digital NPU to a small implementation consisting of 27 MAC PEs to make a fair comparison.

Table 1 compares the area, power consumption, and energy consumption of the ACU chip and the NPU implementations. It can be observed that the proposed ACU chip consumes 85% less chip area and 72.4% less power consumption. For the digital NPU with the full CNN model, it takes 137.9 ms for inference computations through all the layers for a 416 × 416 input image when operating at 200 MHz. After scaling down for the first layer of the CNN model, the digital NPU consumes an average energy of 321.72 μJ. On the other hand, for the case of the proposed ACU chip, it takes 48.5 ms, when operating at 200 MHz, for inference computations of the first layer of the CNN model for a 416 × 416 input image. Therefore, it consumes 26.19 μJ of average energy, which is 92% less than the energy required by the digital NPU. Hence, the proposed analog convolutional operator unit can provide a significant reduction in area, power, and energy consumption compared with its digital counterpart.

Table 2 compares this work with other state-of-the-art analog implementations of convolutional operators. Here, the chip area and power efficiencies are calculated based on the method described in [21]. The mixed-signal cellular neural network presented in [14] realizes the AlexNet CNN model and achieves high computation speed (GOPS). However, it is less energy efficient than other works due to the use of operational transconductance amplifiers as a primary computing unit. The hybrid architecture of [22] incorporates 64 analog convolutional operators integrated with an on-chip CMOS image sensor array for object detection. It occupies a considerable amount of chip area, which is 123× less area efficient than the proposed ACU chip. Its analog convolutional operator constitutes a 4-bit multiplier, which consumes a high power of 18.75 μW, leading to a high energy consumption of 61.98 μJ. In contrast, the proposed MAC unit consumes only 1.44 μW of power, leading to an energy consumption of 26.19 μJ, which makes it 2.5× more energy efficient. The CMOS image sensor integrated with a light-weight CNN presented in [13] consumes less power and thus provides relatively high energy efficiency. However, it suffers from excessively low computation speed due to its low frequency of 2 × 2 kernel operations. Furthermore, it requires excessive chip area as it constitutes 180 computing units, each occupying 4250 μm^2^ of area, integrated with an on-chip pixel memory. The in-memory computing circuit based on capacitors presented in [23] employs an energy recycling technique to achieve high power efficiency. However, our proposed ACU chip area is 2× smaller than [23] when normalized to a 28 nm technology node with the same number of MAC operators. Moreover, the evolving state-of-the-art quantization techniques [20] and the recent low-precision accelerators [22,23] pave the way for analog circuits to operate without requiring high resolutions. The proposed ACU chip demonstrates a relatively smaller chip area and lower power consumption than most of the previous works. Therefore, the proposed architecture is a promising alternative to conventional digital NPUs and previous analog convolutional operator circuits.

## 6. Conclusions

This work proposes a mixed-signal CNN processing chip implementation that aims to replace the digital convolutional units of conventional CNN accelerators. The proposed analog MAC unit comprising symmetric binary-weighted current steering DAC circuits offers a better matching compact and low power consumption design. The proposed 3 × 3 analog convolutional operator unit tightly integrates a MAC unit, a max-pooling circuit, and an ADC to perform convolutional operations. The pooling operation before an ADC in the analog domain reduces the number of ADCs and improves the speed by one-shot convolutional computations. Therefore, the proposed implementation consumes 26.19 μJ of energy, which is 92% less than the fully digital NPU implementation. The presented analog implementation occupies 0.0559 mm^2^ of chip area and consumes 540 μW of power. Hence, the mixed-signal CNN system-on-chip (SoC) promises to be a beneficial replacement as a computing unit in conventional digital CNNs.

## Figures and Tables

**Table 1:** Performance comparison of the proposed analog convolutional operator unit with digital counterpart.

Parameter	Digital NPU [20]		Analog Convolutional Operator Unit		
	288 MAC PEs	27 MAC PEs	27 MAC Units	ADC	Total
Area	3.84 mm^2^	0.384 mm^2^	0.056 mm^2^	0.0046 mm^2^	0.0559 mm^2^
Power	21 mW	1.96 mW	534 μW	6.6 μW	540.6 μW
Energy	2895.9 μJ	321.72 μJ	25.8 μJ	0.31 μJ	26.19 μJ

**Table 2:** Performance comparison with the state-of-the-art works.

Parameter	[14]	[22]	[13]	[23]	This Work
CMOS Technology (nm)	32	65	110	28	55
Target Neural Network	CNN	CNN	CNN	CNN	CNN
Weight Resolution	4	4	-	4	9
Clock Speed (MHz)	-	100	0.398 ^3^	151	200
No. of Computing Units	1024	64	180	32	27
Area of 1 Computing Unit (μm^2^)	-	99,000	4250	943.921	375
Power of 1 Computing Unit (μW)	10	18.75	1.46	-	1.44
ADC Resolution	7-bit SAR	-	4-bit single slope	6-bit SAR	12-Bit SAR
Test Chip Area (mm^2^)	-	15.84	7.65 ^2^	0.031	0.0559
Power Consumption (mW)	-	0.6198	0.96	0.316	0.5406
Computation Speed (GOPS)	251	5.61	0.071	56	5.4
Computation Density (GOPS/mm^2^)	-	0.815	0.0092	1806	96
Power Efficiency (GOPS/W)	-	9060 ^1^	74	177,000 ^1^	9988
Energy Consumption (μJ)	75	61.98	15.36	-	26.19

^1^ Data converted to GOPS. ^2^ Value contains an on-chip pixel array. ^3^ Frequency calculated at 60 fps.

## Data Availability

Data are contained within the article.
